# The association between health behaviours and academic performance moderated by trait mindfulness amongst university students: an observational study

**DOI:** 10.3389/fpubh.2024.1340235

**Published:** 2024-04-19

**Authors:** Sebastian Heller, Jennifer L. Reichel, Lina M. Mülder, Markus Schäfer, Lisa Schwab, Antonia M. Werner, Stephan Letzel, Thomas Rigotti, Pavel Dietz

**Affiliations:** ^1^Institute of Occupational, Social and Environmental Medicine, University Medical Centre, Johannes Gutenberg University Mainz, Mainz, Germany; ^2^Business Psychology and Human Resources, University of Bremen, Bremen, Germany; ^3^Department of Communication, Johannes Gutenberg University Mainz, Mainz, Germany; ^4^Department of Sports Medicine, Rehabilitation and Disease Prevention, Institute of Sport Science, Johannes Gutenberg University Mainz, Mainz, Germany; ^5^Department of Psychosomatic Medicine and Psychotherapy, University Medical Centre, Johannes Gutenberg University Mainz, Mainz, Germany; ^6^Department of Work, Organizational, and Business Psychology, Institute for Psychology, Johannes Gutenberg University Mainz, Mainz, Germany; ^7^Leibniz Institute for Resilience Research, Johannes Gutenberg University Mainz, Mainz, Germany

**Keywords:** health behaviour, academic performance, mindfulness, health communication, health promotion

## Abstract

**Objectives:**

To target health communication at less health-conscious groups, evidence on health behaviours’ effects on non-health-related outcomes – such as academic performance – is necessary. Recent research has highlighted the associations of various health behaviours on academic performance of university students. However, there is a lack of research investigating the most predominant health behaviours simultaneously and their association with academic performance, as well as the factors that potentially influence the direction or strength of these associations. Therefore, this study investigated (I) which of the predominant health behaviours (physical activity, healthy diet, sleep, sedentary behaviour, alcohol consumption, smoking, drug use) are most associated with academic performance and (II) whether the personal resource of trait mindfulness moderates these associations.

**Methods:**

An online survey was conducted amongst university students during the 2021 summer semester. Group differences in academic performance regarding health behaviours were analysed using ANOVA (*N* = 1,049). A first linear regression model (*N* = 571), considering all selected health behaviours simultaneously, assessed their association with academic performance. A second model (*N* = 540) assessed interaction effects of health behaviours and trait mindfulness. Separate regressions assessed each interaction’s association with academic performance.

**Results:**

Sleep, fruit and vegetable consumption, and gender were significantly associated with academic performance. The second model showed no significant interaction effects.

**Conclusion:**

Targeting sleep and fruit and vegetable consumption might be the most promising strategies for elevating students’ academic performance, thereby enabling health communication strategies to reach groups driven by performance improvements rather than health benefits.

## 1 Introduction

One critical setting in presenting health promotion opportunities and challenges is the university (1, 2). In particular, university students represent a unique target group for health promotion because of their likely multiplier role as future professional and social leaders and decision-makers.

Several studies, including systematic reviews and meta-analyses, have examined the prevalence, characteristics, and associated factors of various health behaviours amongst university students, which have recently been summarized in an umbrella review (3). Amongst studies reporting on at least one health behaviour amongst university students, the following are amongst the most commonly studied: one or more of the “big three” health behaviours (4) – physical activity, healthy diet, sleep – and other health behaviours, such as sedentary behaviour, alcohol consumption, smoking, and drug use (5). Each of these health behaviours has an enormous impact on the development of chronic diseases (6–9), although these often only appear as consequences years later. However, other health conditions – particularly mental health conditions – are more directly influenced by health behaviours. In addition, health behaviours are associated with non-health-related outcomes, such as self-esteem (10) and academic performance (5, 11).

From a public health perspective, it is generally important to gather knowledge about as many effects and influences of different health behaviours as possible in order to communicate them to policymakers, stakeholders in higher education and health care, and the public. Therefore, more evidence on the effects of health behaviours on non-health-related outcomes is needed to develop health communication strategies targeted at specific population groups that typically are not motivated by goals of improved health or reduced risk of disease but rather by career- and performance-related improvements, for example. Consequently, for these non-health-oriented population groups, integrating the most effective health behaviour factors that contribute to desirable non-health-related outcomes into health communication strategies may increase their intention to engage in health behaviours by changing their attitudes and beliefs (12). This is possible through media communication, according to theories of health behaviour and communication effects (e.g., the Health Belief Model, the Theory of Reasoned Action, the Theory of Planned Behaviour) (13).

Hence, it is imperative to comprehensively explore the potential correlates (associated factors) of engagement in the most prevalent health behaviours. It is equally important to examine their interactive effects and the respective impacts these health behaviours have on the intended outcomes (14). This investigation is essential to producing significant evidence-based information and developing effective intervention strategies. In this context, *academic performance* appears to be an important non-health-related outcome in the university setting, where it is amongst the most relevant indicators of success. It strongly predicts accomplishments at different educational levels and career outcomes, including job performance and income (15). The concept of *academic performance*, often referred to as “academic achievement” or “academic success” (16), is based, in most countries, on the main indicators of grades and grade point average (GPA) (15).

Over the last decade, various studies have been conducted amongst school-aged children and adolescents, as well as some amongst university or college students, to explore the relationship between various health behaviours and academic performance. This research has led to several systematic reviews that also address the topic, focusing on the associations between academic performance and individual health behaviours, particularly physical activity (17, 18), sedentary behaviour (19), diet (20), or sleep issues (21). Expanding on this area of research, the collection of systematic reviews, which had previously focused on the relationships between academic performance and individual health behaviours, has recently been augmented by a more comprehensive systematic review. This recent study (5) summarized a number of studies on the associations between the most predominant health behaviours and academic performance. A total of 34 studies on diet, physical activity, sedentary behaviour, alcohol consumption, sleep, smoking, and illicit drug use were included in this review. The authors concluded that along with interventions to reduce the negative effects of health behaviours such as insufficient sleep, excessive alcohol consumption, and drug use on academic performance, research is still needed on how health behaviours – and especially their co-occurrence – are related to academic performance. In other words, in addition to examining the most predominant health behaviours simultaneously, examining the factors that potentially influence the direction or strength of the associations between academic performance and health behaviours is essential to gain a deeper understanding of the underlying mechanisms. Of the 34 studies included in this systematic review, only one study amongst Belgian first year students (*N* = 101) assessed all seven predominant health behaviours in association with academic performance (22), and a study amongst Portuguese students (*N* = 1,654) assessed six of these health behaviours (23). The majority of studies included in that review (26 of 34 studies) assessed only one to two health behaviours in association with academic performance.

In the light of the need to examine factors potentially influencing the association between health behaviours and academic performance, the personal resource of *trait mindfulness* appears to be a promising factor for the following reasons: Meditation- and mindfulness-based interventions have spread widely across various settings in the past two decades, becoming a ubiquitous tool in healthcare, corporate health programmes, and educational settings, and a popular subject of scientific investigations across disciplines (24). *Trait mindfulness* (also known as “dispositional mindfulness”) is amongst the most important personality traits for meditation- and mindfulness-based interventions (25). It is characterized by sustained, non-judgmental attention to the present moment (26) and provides a pivotal resource in cognitive control and emotional regulation (27, 28). Consequently, the nuanced interplay between health behaviours and academic performance may be significantly influenced by individual differences in trait mindfulness. For instance, those with higher trait mindfulness might better navigate suboptimal health behaviours’ adverse cognitive and emotional effects, suggesting a potential moderating role. Additionally, in the case of positive health behaviours (e.g., adhering to a healthy diet), individuals with higher trait mindfulness may experience amplified cognitive benefits, further enhancing their academic performance. Examining this moderation can provide a better understanding of the complex dynamics between the personal resource of trait mindfulness, health behaviours, and academic performance, thus paving the way for tailored health and education interventions. Furthermore, previous reviews and meta-analyses have shown the influence of mindfulness practices on academic performance (29), as well as associations between trait mindfulness and health behaviours (30). Given the association between health behaviours and academic performance (5), and also given the relationship between mindfulness and both health behaviours and academic performance, it remains an open question whether trait mindfulness might also (as a moderator) influence the direction and strength of the relationship between health behaviours and academic performance.

To conclude, there is a considerable lack of research that investigated the most predominant health behaviours simultaneously and their association with academic performance. However, particularly analyses of simultaneously occurring predominant health behaviours and their association with academic performance in samples of university students are needed to gain a deeper understanding of which behaviours actually show the strongest association with academic performance. Furthermore, to our knowledge, there is no study that investigated the interplay between the personal resource of trait mindfulness, health behaviours, and academic performance – an examination that is essential to gain a deeper understanding of the underlying mechanisms. Therefore, the present study aimed to investigate (I) which of the predominant health behaviours are most strongly associated with *academic performance* amongst university students, considering all of these health behaviours simultaneously in one model, and (II) whether the potential associations of health behaviours with *academic performance* are moderated by *trait mindfulness*.

## 2 Methods

### 2.1 Study design and survey procedure

During the summer semester of 2021 (June 21–August 15), an online survey was conducted amongst students at the University of Mainz (Germany) as part of an ongoing, evidence-based project on health promotion amongst students (“Healthy Campus Mainz”). The survey was conducted using Unipark software according to the following procedure: the university’s central mailing list was used to invite students to participate via email, thus ensuring a specific, controlled pool of participants. To reduce bias, participation was possible only through the email link sent to all students. Approval to conduct the study was obtained from the Ethics Committee of the Institute of Psychology of Johannes Gutenberg University Mainz (No. 2021-JGU-psychEK-S017).

### 2.2 Measures

The dependent variable *academic performance* was measured by self-reported grade point average (GPA), following Cox et al. (31), as the academic performance of university students is usually indicated by means of GPA (15). The grade range at German universities is 1–5 (a grade ≤ 4 is passing; a grade > 4 is failing). The adapted German question was, “Over the past 12 months, how would you describe your grades in your studies?” Possible responses were “mostly 1.0 or 1.3,” “mostly 1.7, 2.0, or 2.3,” “mostly 2.7, 3.0, or 3.3,” “mostly 3.7, 4.0,” “mostly failed (5.0),” “none of these grades,” and “not sure.” The moderator variable *trait mindfulness* was determined using the 14-item short form of the Freiburg Mindfulness Inventory (FMI) (32). An example item is “I am open to the experience of the present moment.” Response options ranged from “rarely” (1) to “almost always” (4). Cronbach’s α was 0.86.

The selected variables and instruments to measure the health behaviours of physical activity (*moderate-to-vigorous physical activity* [*minutes/week*]), healthy diet (*fruit and vegetable consumption* [*portions/day*]), *sleep (insomnia severity)*, *sedentary behaviour (hours/day)*, *alcohol consumption*, smoking (*smoking cigarettes, e-cigarettes, shisha or cigars*), and drug use (*smoking marijuana* and *pharmacological neuroenhancement*) are displayed in Supplementary Table S1.

### 2.3 Data preparation and analysis

The variables selected for this study were prepared as shown in Supplementary Table S2. To minimize potential bias, the survey data were checked for plausibility using *a priori* criteria, including validation of value ranges, extreme completion times, and implausible responses, such as a reported commitment of more than 50 semester hours per week.

Descriptive statistics are presented as means with standard deviations (SDs) for continuous scaled variables and as numbers and percentages for non-continuous scaled variables. A single-factor analysis of variance (ANOVA) was used to present descriptive statistics and to analyse differences in *academic performance* between the categories of health behaviour variables and *trait mindfulness*. Therefore, *moderate-to-vigorous physical activity (minutes/week)* was categorized according to WHO recommendations (33); *fruit and vegetable consumption (portions/day)* was split at the median (3 portions/day); *sleep (insomnia severity)* was categorized according to the ISI-7 scoring guidelines (34); *sedentary behaviour (hours/day)* was split at the median (8.5 h/day); *alcohol consumption* was categorized at the cut-off score of 4 (35); and *trait mindfulness* was also split at the median (score of 36).

To determine the predictive association of the selected health behaviours with academic performance, a linear regression analysis model was computed with stepwise inclusion of the following sets of variables: the covariates (*age* and *gender*), the big three health behaviours (*moderate-to-vigorous physical activity; fruit and vegetable consumption; sleep (insomnia severity)*); and other health behaviours (*alcohol consumption;* s*moking cigarettes, e-cigarettes, shisha or cigars; smoking marijuana; pharmacological neuroenhancement; sedentary behaviour).* All covariates and health behaviours were simultaneously included in the final step. All continuous scaled variables (*moderate-to-vigorous physical activity*, *fruit and vegetable consumption*, *sleep (insomnia severity)*, *sedentary behaviour*, *alcohol consumption*) were mean-centred (36).

Another linear regression analysis model was computed to determine the predictive association of interaction effects between each health behaviour and the moderator *trait mindfulness* with academic performance. Interaction effect variables were created by multiplying each mean-centred health behaviour variable by the mean-centred moderator *trait mindfulness*. Missings were excluded listwise. To test the robustness of the linear regression models, multicollinearity was checked using the variance inflation factor (VIF) and a correlation matrix of all analysed variables (Supplementary Table S3), and the linear regression models were cross-validated using an 80% random sample (Supplementary Table S4). In addition, to determine the predictive associations of each individual interaction between each health behaviour and *trait mindfulness* with *academic performance*, separate linear regression models were calculated for each interaction. Statistical analyses were performed with IBM SPSS Version 27.

## 3 Results

A total of 1,438 university students participated in the survey. Of these, *N* = 1,049 students responded to the *academic performance* question and were included in the analyses. The mean age of the sample participants was 23.7 (SD = 4.5) years, and 74.1% (*n* = 777) were female. The mean age in the sample was similar to the average age of the entire university student body at that time (24.7 years). Females were overrepresented by 15.1 percentage points. All sociodemographic and study-related characteristics of the participants are listed in Table 1. Regarding participants’ health behaviours, 57.1% (*n* = 414) were highly physically active according to WHO recommendations; mean fruit and vegetable consumption was 3.6 (SD = 2.6) portions/day; 45.3% (*n* = 473) slept without clinically significant insomnia; mean sedentary behaviour was 8.7 (SD = 3.0) hours/day; 30.0% (*n* = 300) reported risky alcohol consumption; 16.2% (*n* = 162) smoked (e)-cigarettes, cigars, or shisha; 7.3% (*n* = 73) smoked marijuana; and 8.8% (*n* = 84) used pharmacological neuroenhancement within the past 12 months. All descriptive statistics for participants’ health behaviours are presented in Table 2.

**Table 1:** Sample characteristics (Healthy Campus Mainz project, Germany, 2021).

Variable	Value
All; *n*	1,049
Gender; *n* (%)	
Female	777 (74.1)
Male	248 (23.7)
Diverse	6 (0.6)
Open	17 (1.6)
Age; range (mean ± SD)	17–69 (23.7 ± 4.5)
Semester; range (mean ± SD)	1–38, (6.8 ± 4.5)
Field of study; *n* (%)	
STEM	170 (17.2)
Social sciences, media or sport	194 (19.7)
Philosophy, humanities or cultural sciences	241 (24.4)
Medicine	141 (14.3)
Law or economics	93 (9.4)
Aspiring teachers	134 (13.6)
Others	13 (1.3)
Degree; *n* (%)	
Bachelor	632 (60.2)
Master	223 (21.3)
State examination	178 (17.0)
Doctorate	6 (0.6)
Other	10 (1.0)
Academic performance; (mean ± SD)	2.1 ± 0.9
Mostly 1.0 or 1.3; *n* (%)	255 (24.3)
Mostly 1.7, 2.0, or 2.3; *n* (%)	503 (48.0)
Mostly 2.7, 3.0, or 3.3; *n* (%)	222 (21.2)
Mostly 3.7, 4.0, or 4.3; *n* (%)	51 (4.9)
Mostly 4.7 or 5.0; *n* (%)	18 (1.7)

**Table 2:** Descriptive statistics for health behaviours, trait mindfulness, and differences regarding academic performance (Healthy Campus Mainz project, Germany, 2021).

Independent study variables	Value	Academic performance; mean ± SD; and its statistical difference between categories of independent variables
Moderate-to-vigorous physical activity (minutes/week); range (mean ± SD)	0–4,620 (463.4 ± 530.0)	*p* = 0.565; *η*^2^ = 0.002
Insufficiently physically active; *n* (%)	189 (26.1)	2.16 ± 0.98
Moderately physically active; *n* (%)	122 (16.8)	2.05 ± 0.90
Highly physically active; *n* (%)	414 (57.1)	2.11 ± 0.83
Fruit and vegetable consumption (portions/day); range (mean ± SD)	2–22 (3.6 ± 2.6)	*p* = 0.002; *η*^2^ = 0.011
Below median (< 3 portion); *n* (%)	568 (65.7)	2.17 ± 0.92
Median and above (≥ 3 portions); *n* (%)	297 (34.3)	1.97 ± 0.81
Sleep (insomnia severity, score: 0–28); mean ± SD	9.3 ± 6.2	*p* < 0.001; *η*^2^ = 0.019
No clinically significant insomnia (0–7); *n* (%)	473 (45.3)	2.01 ± 0.85
Subthreshold insomnia (8–14); *n* (%)	331 (31.7)	2.12 ± 0.87
Clinical insomnia (moderate severity, 15–21); *n* (%)	205 (19.7)	2.33 ± 0.98
Clinical insomnia (severe, 22–28); *n* (%)	34 (3.3)	2.32 ± 0.88
Sedentary behaviour (hours/day); range (mean ± SD)	0–16 (8.7 ± 3.0)	*p* = 0.438; *η*^2^ = 0.001
Below median (< 8.5 h); *n* (%)	501 (52.6)	2.12 ± 0.89
Median and above (≥ 8.5 h); *n* (%)	452 (47.4)	2.08 ± 0.87
Alcohol consumption (score: 0–12); (mean ± SD)	2.5 ± 2.1	*p* = 0.696; *η*^2^ < 0.001
Non-risky consumption (<4); *n* (%)	699 (70.0)	2.12 ± 0.91
Risky Consumption (≥ 4); *n* (%)	300 (30.0)	2.14 ± 0.85
Smoking (e-)cigarettes, cigars or shisha (currently-regularly or occasionally); *n* (%)		*p* = 0.016; *η*^2^ = 0.006
Yes; *n* (%)	162 (16.2)	2.28 ± 0.85
No; *n* (%)	838 (83.8)	2.09 ± 0.90
Smoking marihuana (currently regularly or occasionally); *n* (%)		*p* = 0.582; *η*^2^ < 0.001
Yes; *n* (%)	73 (7.3)	2.07 ± 0.98
No; *n* (%)	928 (92.7)	2.13 ± 0.89
Pharmacological neuroenhancement in the past 12 months; *n* (%)		*p* = 0.010; *η*^2^ = 0.007
Yes; *n* (%)	84 (8.8)	2.36 ± 0.91
No; *n* (%)	870 (91.2)	2.10 ± 0.88
Trait mindfulness (score: 14–56); mean ± SD	35.9 ± 6.8	*p* = 0.020; *η*^2^ = 0.006
Below median (< 36); *n* (%)	508 (53.4)	2.19 ± 0.97
Median and above (≥ 36); *n* (%)	444 (46.6)	2.06 ± 0.79

*p*, statistical significance measured by ANOVA.

### 3.1 Associations of health behaviours with academic performance

Differences in academic performance between categories of health behaviours are presented in Table 2: A*cademic performance* differed significantly between the categories of *fruit and vegetable consumption*, *sleep*, *smoking (e-)cigarettes, cigars or shisha*, and *pharmacological neuroenhancement.* According to the case processing of the linear regression analysis, *n* = 571 cases were included in the analysis. The overall model of the stepwise linear regression (Table 3, Model 1), including covariates (*age* and *gender*) and all health behaviours to predict the dependent variable *academic performance,* was statistically significant. Multicollinearity testing revealed no collinearity of the selected variables (average VIF = 1.12 [1.01–1.28]). The explained variance (Nagelkerke R^2^) in the final step of the four-step model was 2.8% (see Table 3). In the previous steps, the explained variance was 0.7% in Step 1 (covariates *age* and *gender* only), 2.2% in Step 2 (covariates and big three health behaviours – *moderate-to-vigorous physical activity*, *fruit and vegetable consumption*, *sleep*), and 1.6% in Step 3 (covariates and other health behaviours – *alcohol consumption*, *smoking*, *marijuana smoking*, *pharmacological neuroenhancement*, *sedentary behaviour*). Cross-validation with an 80% random sample showed the robustness of the regression model (see Supplementary Tables S4).

**Table 3:** Linear regression analysis of academic performance with stepwise inclusion of health behaviours and health behaviours’ interaction with trait mindfulness (Healthy Campus Mainz project, Germany, 2021).

	Model 1: covariates and health behaviours to predict academic performance												Model 2: covariates, health behaviours, trait mindfulness (TM), and interactions between health behaviours and TM to predict academic performance		
	Step 1: covariates (*R*^2^ = 0.007*)			Step 2: big three health behaviours (*R*^2^ = 0.022***)			Step 3: other health behaviours (*R*^2^ = 0.016)			Step 4: all health behaviours (*R*^2^ = 0.028**)			(*R*^2^ = 0.026*)		
	B (SE)	*β*	*p*	B (SE)	*β*	*p*	B (SE)	*β*	*p*	B (SE)	*β*	*p*	B (SE)	*β*	*p*
Gender “female”	−0.17 (0.07)	−0.08	0.009	−0.17 (0.09)	−0.05	0.208	−0.13 (0.07)	−0.06	0.061	−0.09 (0.09)	−0.05	0.304	−0.11 (0.09)	−0.05	0.229
Gender “diverse”	0.42 (0.37)	0.04	0.250	−0.17 (0.52)	−0.01	0.744	0.58 (0.44)	0.04	0.193	0.12 (0.51)	−0.01	0.823	−0.04 (0.51)	<−0.01	0.943
Gender “open”	−0.43 (0.23)	−0.06	0.059	−0.26 (0.29)	−0.04	0.367	−0.49 (0.26)	−0.06	0.060	−0.64 (0.32)	−0.09	0.047	−0.54 (0.35)	−0.07	0.128
Age	<−0.01 (<0.01)	−0.03	0.363	<−0.01 (<0.01)	−0.01	0.824	−0.01 (<0.01)	−0.05	0.161	<−0.01 (<0.01)	−0.04	0.340	−0.08 (0.08)	−0.04	0.332
Moderate-to-vigorous physical activity (minutes/week)				<0.01 (<0.01)	0.06	0.163				<0.01 (<0.01)	0.05	0.244	<0.01 (<0.01)	0.05	0.227
Fruit and vegetables consumption (portions/day)				−0.03 (0.01)	−0.09	0.019				−0.04 (0.01)	−0.11	0.011	−0.04 (0.02)	−0.11	0.014
Sleep (insomnia severity)				0.13 (0.04)	0.13	0.001	0.12 (0.04)	0.12	0.004	0.14 (0.05)	0.13	0.003
Sedentary behaviour (hours/day)							−0.01 (0.01)	−0.03	0.446	<−0.01 (0.01)	−0.01	0.782	<−0.01 (0.01)	<−0.01	0.910
Alcohol consumption (AUDIT-C score)							<−0.01 (0.05)	<−0.01	0.954	−0.03 (0.06)	−0.02	0.682	−0.01 (0.06)	−0.01	0.854
Smoking (currently: no/yes)							0.16 (0.09)	0.07	0.065	0.19 (0.11)	0.08	0.079	0.16 (0.12)	0.06	0.166
Smoking marihuana (currently: no/yes)							−0.18 (0.13)	−0.05	0.147	−0.05 (0.15)	−0.02	0.721	−0.03 (0.15)	−0.01	0.837
Pharmacological neuroenhancement (past 12 months: no/yes)							0.27 (0.11)	0.09	0.019	0.09 (0.15)	0.03	0.553	0.09 (0.15)	0.03	0.566
Trait mindfulness													<−0.01 (0.09)	<−0.01	0.966
TM * Moderate-to-vigorous physical activity (minutes/week)													<0.01 (<0.01)	0.02	0.713
TM * Fruit and vegetables consumption (portions/day)													0.04 (0.03)	0.06	0.218
TM * Sleep (insomnia severity)													0.04 (0.10)	0.02	0.646
TM * Sedentary behaviour (hours/day)													−0.03 (0.03)	−0.04	0.332
TM * Alcohol Consumption													−0.22 (0.13)	−0.08	0.093
TM * Smoking													−0.35 (0.92)	−0.02	0.700
TM * Pharmacological neuroenhancement													0.18 (0.38)	0.02	0.643
TM * Smoking marihuana													−0.22 (0.43)	−0.02	0.612

*p < 0.05; **p < 0.01; ***p < 0.001. Model 1: Step 1: F(4, 1,042) = 2.960, *p* = 0.019; Step 2: F(7, 621) = 3.045, *p* = 0.004; Step 3: F(9, 898) = 2.592, *p* = 0.006; Step 4: F(12, 571) = 2.412, *p* = 0.005. Model 2: F(21, 540) = 1.721, *p* = 0.024. TM*, trait mindfulness interactions with independent variables.

The fourth step of the regression – including covariates and all health behaviours (the big three and other health behaviours) – showed that *sleep (insomnia severity)* (*β* = 0.12*, p* = 0.004), *fruit and vegetable consumption* (*β* = −0.11*, p* = 0.011), and *gender “open”* (*β* = −0.09*, p* = 0.047) were significantly related to *academic performance*. Of the other health behaviour variables, only *pharmacological neuroenhancement* showed a significant association with *academic performance* in the previous Step 3 but not in the final (4th) step of the linear regression model (see Table 3).

### 3.2 Health behaviours, trait mindfulness and their interaction in association with academic performance

ANOVA showed that *academic performance* was significantly higher amongst participants with higher *trait mindfulness* (above the median; see Table 2). For the linear regression model predicting *academic performance* by including covariates (age and gender), all health behaviours, *trait mindfulness,* and interactions between each health behaviour and *trait mindfulness* (Model 2; see Table 3), *n* = 540 cases were included in the analysis. Linear regression Model 2 was statistically significant. The multicollinearity inspection revealed no collinearity of the selected variables (average VIF = 1.19 [1.01–1.32]). The explained variance (Nagelkerke R^2^) was 2.6% (see Table 3). However, note that when regression Model 2 was cross-validated with an 80% random sample, the model was no longer statistically significant (see Supplementary Table S4).

In the original sample model, *sleep (insomnia severity)* (*β* = 0.13*, p* = 0.004) and *fruit and vegetable consumption (portions/day)* (*β* = −0.11*, p* = 0.014) were significantly associated with *academic performance*. There was no significant interaction effect between any health behaviour and *trait mindfulness*, and no significant association of any health behaviour or *trait mindfulness* with *academic performance* (see Table 3).

One noteworthy outcome emerged concerning the distinct linear regression models that incorporated individual health behaviours, trait mindfulness, and their corresponding interactions. Specifically, the model encompassing alcohol consumption exhibited statistical significance, standing out from the rest (*F*[3, 947] = 3.051, *p* = 0.028). The variance explained by this model was 0.6%. The interaction between alcohol consumption and trait mindfulness proved to be the only statistically significant predictor (*β* = −0.08, *p* = 0.018). However, it is noteworthy that the individual variables of alcohol consumption (*β* = 0.04, *p* = 0.185) and trait mindfulness (*β* = −0.06, *p* = 0.087) did significantly relate to academic performance on their own.

## 4 Discussion

The present study investigated the predictive impact of health behaviours on academic performance amongst university students, as well as the potential moderating role of trait mindfulness. The results indicate that when the most predominantly studied health behaviours are considered simultaneously, only sleep and fruit and vegetable consumption stand out as being associated with students’ academic performance. In addition, based on the present results, trait mindfulness does not appear to play a critical role in influencing the direction or strength of the association between health behaviours and academic performance.

Regarding the first research objective, the result that only sleep and fruit and vegetable consumption were significantly related to academic performance is surprising: one might have expected the big three health behaviours (sleep, fruit and vegetable consumption, and moderate-to-vigorous physical activity) to be related to academic performance. Thus, it is noteworthy that only these two had any significant impact and that physical activity showed no correlation with academic performance. Furthermore, the variance explained by health behaviours in academic performance is notably lower than in other studies that examined only certain health behaviours concurrently. For instance, Kristjánsson et al. (37) explained 36% of the variance in academic performance by sedentary lifestyle, body mass index, and physical activity, while Busch et al. (38) explained 27% of the variance in girls and 15% in boys by alcohol consumption, cannabis consumption, smoking, screen time, bullying/being bullied, healthy diet, and physical activity. However, it is essential to consider that the samples in those studies were composed of school-aged children and adolescents from Iceland (37) and the Netherlands (38), which differ significantly from our sample in age, life stage, and educational system. Furthermore, the present survey was conducted during the COVID-19 pandemic, a period marked by substantial disruptions in daily routines and lifestyles, leading to significant changes in various health behaviours (39–43). This context makes it challenging to distinguish between pandemic-induced behaviours and inherent student population behaviours. The unique pandemic circumstances could also have impacted the association between health behaviours and academic performance, which was measured as a snapshot during this period. It is important to mention that academic performance, assessed through GPA, may also reflect pre-pandemic performance, since the participants were advanced university students with grades earned before the pandemic. This factor could potentially complicate the observed associations in the present study and contribute to the lower explained variance in academic performance.

Interestingly, no significant association (not even a single association by ANOVA) between moderate-to-vigorous physical activity and academic performance was detected in this study, in contrast to previous studies that reported strong associations between these constructs (44). One possible explanation for this discrepancy may be that the sample studied appeared to be quite physically active, as indicated by the relatively large proportion (57%) that was classified as “highly physically active” according to WHO recommendations (33). This large proportion is particularly striking when compared with data on student samples from other studies conducted during the COVID-19 pandemic in 2021 (e.g., 10.5% in Italian students) (45) but it is roughly in line with figures from previous health surveys at the same university (60% highly physically active in 2019) (46).

A valuable ancillary finding was the significant association of pharmacological neuroenhancement with lower academic performance (in Step 3 of regression Model 1 and ANOVA results). This adds interesting evidence to the scientific discussion of whether pharmacological neuroenhancement is associated with better grades (47).

Regarding the second research aim, this study stands as the first to explore the potential interaction effect of health behaviours and trait mindfulness. However, only during additional analysis, where separate regression models were computed, focusing on individual health behaviours, trait mindfulness, and their interactions, did we observe a significant interaction between alcohol consumption and trait mindfulness, linked to academic performance. This interaction indicated that higher trait mindfulness was associated with better academic performance, particularly lowering the negative association of alcohol consumption with academic performance. Yet, it should be noted that the effects were relatively small; thus, the influence of trait mindfulness on the association between health behaviours and academic performance must be interpreted with caution. Additionally, it is crucial to highlight that we focused on trait mindfulness rather than mindfulness practice. Given the results of this cross-sectional analysis and considering existing systematic reviews and meta-analyses showing separate associations between health behaviours, trait mindfulness or mindfulness practices, and academic performance (5, 29, 30), future research could explore potential mediating rather than moderating effects of trait mindfulness on health behaviours and their associations with academic performance in longitudinal study designs.

Regarding the study limitations, due to the cross-sectional design, it was not possible to investigate any causal relationships between the variables examined. Therefore, as mentioned above, longitudinal studies would be highly enriching. Furthermore, we did not directly inquire about the presence of learning disabilities amongst participants, a factor that could significantly influence the associations and interactions between academic performance, health behaviours, and trait mindfulness. Regarding diversity and the generalizability of our study results, the overrepresentation of female participants in our sample, compared to the university’s overall student body, may limit their applicability to other (student) populations. In addition, although the student participants came from different fields of study, the study sample seemed relatively health-conscious. This was indicated, for example, by the relatively high proportion of highly physically active students. This potential source of bias in our analyses, in addition to the previously mentioned bias due to the impact of the COVID-19 pandemic on health behaviours, may have also reduced the variability in the expression of health behaviours and thus the likelihood of detecting an association with academic performance. Furthermore, an appropriately larger sample size may have yielded more robust results – particularly for linear regression Model 2 (including covariates, health behaviours, trait mindfulness, and the interaction effects of health behaviours and trait mindfulness).

## 5 Conclusion

Our results provide novel and valuable insights into the trend that strategies focusing on sleep and fruit and vegetable consumption may be the most promising to address students’ academic performance. In this context, we emphasize that particularly from a public health perspective, academic performance can be seen as a means to a higher end: improving health and well-being and contributing to a healthier lifestyle. Therefore, academic performance could be a motivational goal expectation for health behaviour change for use in health communication strategies to reach those groups (not only students but also decision-makers and stakeholders in higher education) who may not typically feel inspired by health improvement goals or reducing the risk of disease but perhaps more by career- and performance-related improvements.

## Data availability statement

The raw data supporting the conclusions of this article will be made available by the authors, without undue reservation.

## Ethics statement

The studies involving humans were approved by Ethics Committee of the Institute of Psychology of Johannes Gutenberg University Mainz. The studies were conducted in accordance with the local legislation and institutional requirements. The participants provided their written informed consent to participate in this study.

## Author contributions

SH: Conceptualization, Data curation, Formal analysis, Investigation, Methodology, Software, Validation, Writing – original draft, Writing – review & editing. JR: Data curation, Writing – review & editing. LM: Data curation, Writing – review & editing. MS: Data curation, Writing – review & editing. LS: Data curation, Writing – review & editing. AW: Data curation, Writing – review & editing. SL: Funding acquisition, Project administration, Resources, Writing – review & editing. TR: Conceptualization, Data curation, Formal analysis, Methodology, Software, Validation, Writing – review & editing. PD: Conceptualization, Data curation, Formal analysis, Investigation, Methodology, Project administration, Resources, Software, Supervision, Validation, Writing – review & editing.

## References

[1] International Conference on Health Promoting Universities & Colleges. Okanagan charter: An international charter for health promoting universities and colleges: An outcome of the 2015 international conference on health promoting Universitites and colleges / VII international congress. Kelowna, Canada (2015).

[2] WHO. Ottawa Charter for Health Promotion [internet]. World Health Organization. Geneva: World Health Organization (1986). Available from: https://www.euro.who.int/__data/assets/pdf_file/0006/129534/Ottawa_Charter_G.pdf.

[3] Dietz P Reichel JL Edelmann D Werner AM Tibubos AN Schäfer M. A systematic umbrella review on the epidemiology of modifiable health influencing factors and on health promoting interventions among university students. Front Public Health. (2020) 8:137. doi: 10.3389/fpubh.2020.00137, PMID: 32411645 PMC7198755

[4] Wickham S-R Amarasekara NA Bartonicek A Conner TS. The big three health Behaviors and mental health and well-being among young adults: a cross-sectional investigation of sleep, exercise, and diet. Front Psychol. (2020) 11:579205. doi: 10.3389/fpsyg.2020.579205, PMID: 33362643 PMC7758199

[5] Whatnall M Ashton L Patterson A Smith J Duncan M Burrows T. Are health behaviors associated with academic performance among tertiary education students? A systematic review of cohort studies. J Am Coll Heal. (2022):1–13. doi: 10.1080/07448481.2022.2063024, PMID: 35549627

[6] Patterson R McNamara E Tainio M de Sá TH Smith AD Sharp SJ. Sedentary behaviour and risk of all-cause, cardiovascular and cancer mortality, and incident type 2 diabetes: a systematic review and dose response meta-analysis. Eur J Epidemiol. (2018) 33:811–29. doi: 10.1007/s10654-018-0380-1, PMID: 29589226 PMC6133005

[7] Ekelund U Tarp J Steene-Johannessen J Hansen BH Jefferis B Fagerland MW. Dose-response associations between accelerometry measured physical activity and sedentary time and all cause mortality: systematic review and harmonised meta-analysis. BMJ. (2019) 366:l4570. doi: 10.1136/bmj.l4570, PMID: 31434697 PMC6699591

[8] Yin J Jin X Shan Z Li S Huang H Li P. Relationship of sleep duration with all-cause mortality and cardiovascular events: a systematic review and dose-response meta-analysis of prospective cohort studies. J Am Heart Assoc. (2017) 6:6. doi: 10.1161/JAHA.117.005947, PMID: 28889101 PMC5634263

[9] GBD 2019 Diseases and Injuries Collaborators. Global burden of 369 diseases and injuries in 204 countries and territories, 1990-2019: a systematic analysis for the global burden of disease study 2019. Lancet. (2020) 396:1204–22. doi: 10.1016/S0140-6736(20)30925-9 33069326 PMC7567026

[10] Arsandaux J Montagni I Macalli M Bouteloup V Tzourio C Galéra C. Health risk Behaviors and self-esteem among college students: systematic review of quantitative studies. Int J Behav Med. (2020) 27:142–59. doi: 10.1007/s12529-020-09857-w, PMID: 32072455

[11] Busch V Loyen A Lodder M Schrijvers AJ van Yperen TA De LJR. The effects of adolescent health-related behavior on academic performance. Rev Educ Res. (2014) 84:245–74. doi: 10.3102/0034654313518441

[12] Montaño DE Kasprzyk D. Theory of reasoned action, theory of planned behavior, and the integrated behavioral model In: Glanz K Rimer BK Viswanath KV, editors. Health behavior: Theory, research, and practice. 5th ed. Hoboken, NJ, US: Jossey-Bass/Wiley (2015). 95–124.

[13] Viswanath K Finnegan JR Jr Gollust S. Communication and health behavior in a changing media environment In: Glanz K Rimer BK Viswanath KV, editors. Health behavior: Theory, research, and practice. 5th ed. Hoboken, NJ, US: Jossey-Bass/Wiley (2015). 327–48.

[14] Miettinen OS. Important concepts in epidemiology In: Olsen J Saracci R Trichopoulos D, editors. Teaching epidemiology. Oxford: Oxford University Press (2010). 25–50.

[15] Kuncel NR Credé M Thomas LL. The validity of self-reported grade point averages, class ranks, and test scores: a meta-analysis and review of the literature. Rev Educ Res. (2005) 75:63–82. doi: 10.3102/00346543075001063

[16] York TT Gibson C Rankin S. Defining and measuring academic success. Practical Research, Assessment & Evaluation. (2015) 20.

[17] Peiris DL Duan Y Vandelanotte C Liang W Yang M Baker JS. Effects of in-classroom physical activity breaks on Children's academic performance, cognition, health behaviours and health outcomes: a systematic review and meta-analysis of randomised controlled trials. Int J Environ Res Public Health. (2022) 19:19. doi: 10.3390/ijerph19159479, PMID: 35954831 PMC9368257

[18] Watson A Timperio A Brown H Best K Hesketh KD. Effect of classroom-based physical activity interventions on academic and physical activity outcomes: a systematic review and meta-analysis. Int J Behav Nutr Phys Act. (2017) 14:114. doi: 10.1186/s12966-017-0569-9, PMID: 28841890 PMC5574081

[19] Minges KE Chao AM Irwin ML Owen N Park C Whittemore R. Classroom standing desks and sedentary behavior: a systematic review. Pediatrics. (2016) 137:e20153087. doi: 10.1542/peds.2015-3087, PMID: 26801914 PMC4732360

[20] Burrows T Goldman S Pursey K Lim R. Is there an association between dietary intake and academic achievement: a systematic review. J Hum Nutr Diet. (2017) 30:117–40. doi: 10.1111/jhn.12407, PMID: 27599886

[21] Suardiaz-Muro M Morante-Ruiz M Ortega-Moreno M Ruiz MA Martín-Plasencia P Vela-Bueno A. Sueño y rendimiento académico en estudiantes universitarios: revisión sistemática. Rev Neurol. (2020) 71:43–53. doi: 10.33588/rn.7102.2020015 32627159

[22] Deliens T Clarys P de Bourdeaudhuij I Deforche B. Weight, socio-demographics, and health behaviour related correlates of academic performance in first year university students. Nutr J. (2013) 12:162. doi: 10.1186/1475-2891-12-162, PMID: 24344995 PMC3878497

[23] Gomes AA Tavares J de Azevedo MH. Sleep and academic performance in undergraduates: a multi-measure, multi-predictor approach. Chronobiol Int. (2011) 28:786–801. doi: 10.3109/07420528.2011.606518, PMID: 22080785

[24] van Dam NT van Vugt MK Vago DR Schmalzl L Saron CD Olendzki A. Mind the hype: a critical evaluation and prescriptive agenda for research on mindfulness and meditation. Perspect Psychol Sci. (2018) 13:36–61. doi: 10.1177/1745691617709589, PMID: 29016274 PMC5758421

[25] Tang Y-Y Tang R. Personality and meditation. Neurosci Meditation. (2020):15–36. doi: 10.1016/b978-0-12-818266-6.00003-4

[26] Kabat-Zinn J. Mindfulness-based interventions in context: past, present, and future. Clin Psychol Sci Pract. (2003) 10:144–56. doi: 10.1093/clipsy.bpg016

[27] Mesmer-Magnus J Manapragada A Viswesvaran C Allen JW. Trait mindfulness at work: a meta-analysis of the personal and professional correlates of trait mindfulness. Hum Perform. (2017) 30:79–98. doi: 10.1080/08959285.2017.1307842

[28] Prakash RS Hussain MA Schirda B. The role of emotion regulation and cognitive control in the association between mindfulness disposition and stress. Psychol Aging. (2015) 30:160–71. doi: 10.1037/a0038544, PMID: 25545683

[29] Ostermann T Pawelkiwitz M Cramer H. The influence of mindfulness-based interventions on the academic performance of students measured by their GPA. A systematic review and meta-analysis. Front. Behav Neurosci. (2022) 16:961070. doi: 10.3389/fnbeh.2022.961070, PMID: 36090656 PMC9462381

[30] Sala M Rochefort C Lui PP Baldwin AS. Trait mindfulness and health behaviours: a meta-analysis. Health Psychol Rev. (2020) 14:345–93. doi: 10.1080/17437199.2019.1650290, PMID: 31362588

[31] Cox RG Zhang L Johnson WD Bender DR. Academic performance and substance use: findings from a state survey of public high school students. J Sch Health. (2007) 77:109–15. doi: 10.1111/j.1746-1561.2007.00179.x, PMID: 17302852

[32] Walach H Buchheld N Buttenmüller V Kleinknecht N Grossmann P Schmidt S. Empirische Erfassung der Achtsamkeit - Die Konstruktion des Freiburger Fragebogens zur Achtsamkeit (FFA) und weitere Validierungsstudien In: Heidenreich T, editor. Achtsamkeit und Akzeptanz in der Psychotherapie: Ein Handbuch. Tübingen: DGVT Deutsche Gesellschaft f. Verhaltenstherapie (2004). 727–65.

[33] World Health Organization. WHO guidelines on physical activity and sedentary behaviour. Geneva: World Health Organization (2020). 93 p. 33369898

[34] Gerber M Lang C Lemola S Colledge F Kalak N Holsboer-Trachsler E. Validation of the German version of the insomnia severity index in adolescents, young adults and adult workers: results from three cross-sectional studies. BMC Psychiatry. (2016) 16:174. doi: 10.1186/s12888-016-0876-8, PMID: 27245844 PMC4888604

[35] Bush K Kivlahan DR McDonell MB Fihn SD Bradley KA. The AUDIT alcohol consumption questions (AUDIT-C): an effective brief screening test for problem drinking. Ambulatory care quality improvement project (ACQUIP). Alcohol use disorders identification test. Arch Intern Med. (1998) 158:1789–95. doi: 10.1001/archinte.158.16.1789, PMID: 9738608

[36] Hofer M. Mean Centering In: Matthes J Davis CS Potter RF, editors. The international Encyclopedia of communication research methods. Hoboken, NJ: Wiley (2017). 1–3.

[37] Kristjánsson AL Sigfúsdóttir ID Allegrante JP Helgason AR. Adolescent health behavior, contentment in school, and academic achievement. Am J Health Behav. (2009) 33:69–79. doi: 10.5993/AJHB.33.1.7 18844522

[38] Busch V Laninga-Wijnen L Schrijvers AJ de Leeuw JR. Associations of health behaviors, school performance and psychosocial problems in adolescents in the Netherlands. Health Promot Int. (2017) 32:280–91. doi: 10.1093/heapro/dav058, PMID: 26094252

[39] Bakaloudi DR Evripidou K Siargkas A Breda J Chourdakis M. Impact of COVID-19 lockdown on smoking and vaping: systematic review and meta-analysis. Public Health. (2023) 218:160–72. doi: 10.1016/j.puhe.2023.02.007, PMID: 37043948 PMC9939396

[40] Roberts A Rogers J Mason R Siriwardena AN Hogue T Whitley GA. Alcohol and other substance use during the COVID-19 pandemic: a systematic review. Drug Alcohol Depend. (2021) 229:109150. doi: 10.1016/j.drugalcdep.2021.109150, PMID: 34749198 PMC8559994

[41] Deng J Zhou F Hou W Silver Z Wong CY Chang O. The prevalence of depressive symptoms, anxiety symptoms and sleep disturbance in higher education students during the COVID-19 pandemic: a systematic review and meta-analysis. Psychiatry Res. (2021) 301:113863. doi: 10.1016/j.psychres.2021.113863, PMID: 33984824 PMC9225824

[42] Jehi T Khan R Halawani R Dos Santos H. Effect of COVID-19 outbreak on the diet, body weight and food security status of students of higher education: a systematic review. Br J Nutr. (2023) 129:1916–28. doi: 10.1017/S0007114522002604, PMID: 35946073

[43] Park AH Zhong S Yang H Jeong J Lee C. Impact of COVID-19 on physical activity: a rapid review. J Glob Health. (2022) 12:5003. doi: 10.7189/jogh.12.05003, PMID: 35493780 PMC8979477

[44] Wunsch K Fiedler J Bachert P Woll A. The Tridirectional relationship among physical activity, stress, and academic performance in university students: a systematic review and meta-analysis. Int J Environ Res Public Health. (2021) 18:18. doi: 10.3390/ijerph18020739, PMID: 33467118 PMC7830011

[45] Roggio F Trovato B Ravalli S Di Rosa M Maugeri G Bianco A. One year of COVID-19 pandemic in Italy: effect of sedentary behavior on physical activity levels and musculoskeletal pain among university students. Int J Environ Res Public Health. (2021) 18:18. doi: 10.3390/ijerph18168680, PMID: 34444427 PMC8392636

[46] Edelmann D Pfirrmann D Heller S Dietz P Reichel JL Werner AM. Physical activity and sedentary behavior in university students-the role of gender, age, Field of study, targeted degree, and study semester. Front Public Health. (2022) 10:821703. doi: 10.3389/fpubh.2022.821703, PMID: 35784227 PMC9244168

[47] Arria AM Caldeira KM Vincent KB O'Grady KE Cimini MD Geisner IM. Do college students improve their grades by using prescription stimulants nonmedically? Addict Behav. (2017) 65:245–9. doi: 10.1016/j.addbeh.2016.07.016, PMID: 27469455 PMC5140739

