# Supplementary material for: The association between health behaviours and academic performance moderated by trait mindfulness amongst university students: an observational study
Source: Front Public Health. 2024 Apr 19;12:1340235. doi: 10.3389/fpubh.2024.1340235 (PMC11066253; doi:10.3389/fpubh.2024.1340235)
Supplement: Supplementary file 1 [file Table_1.PDF]

Supplementary data for "The association between health behaviours and academic performance moderated by trait mindfulness among university students: An observational study"

Supplementary Table S1: Variables and instruments used to measure health behaviours

**Health behaviour**	**Variable**	**Instrument**
Physical activity	*Moderate-to-vigorous physical activity (minutes/week)*	The short-form International Physical Activity Questionnaire (IPAQ-SF) (1), a validated and reliable instrument (2), was used to determine *moderate-to vigorous physical activity (minutes/week).*
Healthy diet	*Fruit and vegetable consumption (portions/day)*	For the assessment of a healthy diet, the key component (3, 4) *fruit and vegetable consumption (portions/day)* was chosen and measured using of the items from the European Health Interview Survey 2013 (5).
Sleep	*Sleep (insomnia severity)*	It was determined by using the Isomnia Severity Index (ISI-7) (6), a brief instrument to assess respondents' perceptions of sleep complaints.
Sedentary behaviour	*Sedentary behaviour (hours/day)*	The short-form International Physical Activity Questionnaire (IPAQ-SF) (1), a validated and reliable instrument (2), was used to determine *sedentary behaviour.*
Alcohol consumption	*Alcohol consumption*	It was determined using the "Alcohol-Use-Disorder-Identification-Test Consumption" (AUDIT-C) which is the 3-item short version of the original 10-item questionnaire published by the World Health Organization (7). Throughout many studies, the AUDIT-C has emerged as a simple instrument to measure risky alcohol consumption, also among students (8, 9).
Smoking	*Smoking cigarettes, e-cigarettes, shisha or cigars*	It was included as a variable summarizing common smoking products and surveyed according to Töpritz et al. (10). The survey contained questions regarding the frequency of consumption separately for each smoking type. The translated question was "Do you smoke [respective smoking type]?" Possible responses were "never", "former occasionally", "former regularly", "currently occasionally", or "currently regularly".
Drug use	*Smoking marihuana*	It was included as a variable representing the most common illicit drug use among students and surveyed according to Töpritz et al. (10). The survey contained questions regarding the frequency of consumption separately for each smoking type. The translated question was "Do you smoke [respective smoking type]?" Possible responses were "never", "former occasionally", "former regularly", "currently occasionally", or "currently regularly".
	Pharmacological neuroenhancement within the past 12 months	It was chosen to include the variable *pharmacological neuroenhancement within the past 12 months* as a form of misusing of drugs that is particularly prevalent among the target group of university students (11). Pharmacological neuroenhancement is defined as the use of illicit or prescription drugs by healthy individuals for cognitive-enhancing purposes (12–14), such as enhancing alertness, attention, concentration, memory, and also mood (15, 16). It was surveyed according to Dietz et al. (15).

Note: The references can be found in the last section of this document

Supplementary Table S2: Data preparation of the variables used for study analyses.

Variable	Data preparation
Academic performance	To enable analyses only with valid grades, participants who responded 'none of these grades' or 'not sure' were excluded.
Moderate-to-vigorous physical activity (minutes/week)	It was computed according to the IPAQ data processing guidelines (17) and by using a spreadsheet from Cheng et al. (18).
Fruit and vegetable consumption (portions/day)	It was computed by summing up the individual figures for portions of fruit and portions of vegetables per day.
Sleep (insomnia severity)	It was computed according to the scoring of the insomnia severity index (ISI-7) (6).
Sedentary behaviour (hours/day)	It was computed according to the IPAQ data processing guidelines (17) and by using a spreadsheet from Cheng et al. (18).
alcohol consumption	It was scored according to the AUDIT-C guidelines (9).
Smoking cigarettes, e-cigarettes, shisha or cigars	In order to calculate the analyses with current smokers and non-smokers, the variable was dichotomized: For currently smoking the respective type, we defined "currently occasionally" and "currently regularly" as "Yes", and "never", "former occasionally", "former regularly" as "No".
Smoking marihuana	In order to calculate the analyses with current smokers and non-smokers, the variable was dichotomized: For currently smoking marihuana, we defined "currently occasionally" and "currently regularly" as "Yes", and "never", "former occasionally", "former regularly" as "No".
Pharmacological neuroenhancement within the past 12 months	In order to enable the investigation of potential changes in the past 12-month use of pharmacological neuroenhancement in future studies, the use within the past 12 months instead of the use within a lifetime was chosen for all analyses. Therefore, the variable was dichotomized (used within the last 12 months/never used PN) and the participants who answered 'more than 12 months ago' were excluded – according to Heller et al. (19).
Trait mindfulness	It was formed by summing the 14 items of the FMI (20), with one inverted item (item 29: "I am impatient with myself and those around me") first being reversed and then summed.

Note: The references can be found in the last section of this document

Supplementary Table S3: Correlation matrix including all independent study variables and the moderator variable.

		1	2	3	4	5	6	7	8	9	10	11	12	13	14	15	16	17	18	19	20	21
1) Gender “female”	Pearson-Korrelation	--																				
2) Gender “diverse”	Pearson-Korrelation	-,128**	--																			
	Sig. (2-seitig)	.0																				
3) Gender “open”	Pearson-Korrelation	-,217**	-.010	--																		
	Sig. (2-seitig)	.0	.753																			
4) Age	Pearson-Korrelation	-.022	.005	.013	--																	
	Sig. (2-seitig)	.479	.881	.681																		
5) Moderate-to-vigorous physical activity (minutes/week)	Pearson-Korrelation	.009	.013	-.053	-.002	--																
	Sig. (2-seitig)	.803	.729	.154	.949																	
6) Fruit and vegetables consumption (portions/day)	Pearson-Korrelation	,071*	.037	-.026	.006	.059	--															
	Sig. (2-seitig)	.036	.275	.446	.867	.136																
7) Sleep (insomnia severity)	Pearson-Korrelation	.040	,066*	,074*	-.033	.014	-.025	--														
	Sig. (2-seitig)	.193	.032	.018	.288	.699	.470															
8) Sedentary behavior (hours/day)	Pearson-Korrelation	-.009	.015	.035	-.019	-,248**	.053	,069*	--													
	Sig. (2-seitig)	.784	.640	.280	.556	.0	.127	.033														
9) Alcohol consumption (AUDIT-C score)	Pearson-Korrelation	-,116**	-.025	-.024	-.028	,095*	-.058	.014	-,080*	--												
	Sig. (2-seitig)	.0	.438	.447	.379	.010	.088	.661	.013													
10) Smoking (currently: no/yes)	Pearson-Korrelation	-,085**	.001	-.016	.006	.061	-.066	.052	-.041	,357**	--											
	Sig. (2-seitig)	.008	.976	.616	.842	.099	.054	.099	.204	.0												
11) Smoking marihuana (currently: no/yes)	Pearson-Korrelation	-.045	.028	.053	-.018	.032	-.016	-.003	-.060	,170**	,203**	--										
	Sig. (2-seitig)	.153	.369	.093	.579	.394	.642	.916	.066	.0	.0											
12) Pharmacological neuroenhancement (past 12 months: no/yes)	Pearson-Korrelation	-,089**	,069*	-.007	.002	.044	-.014	,113**	-.014	,196**	,235**	,403**	--									
	Sig. (2-seitig)	.006	.034	.824	.945	.247	.692	.001	.668	.0	.0	.0										
13) Trait mindfulness	Pearson-Korrelation	-.029	-,077*	-,066*	.021	,102**	.052	-,272**	-.064	,093**	.012	.005	-.017	--								
	Sig. (2-seitig)	.372	.018	.041	.518	.007	.132	.0	.055	.004	.706	.889	.610									
14) TM * Moderate-to-vigorous physical activity (minutes/week)	Pearson-Korrelation	.005	.034	.034	-.001	-.042	.019	-.068	-.049	.046	-.013	-.005	-.029	,155**	--							
	Sig. (2-seitig)	.889	.366	.365	.982	.271	.635	.076	.202	.223	.725	.902	.448	.0								
15) TM * Fruit and vegetables consumption (portions/day)	Pearson-Korrelation	.044	-.003	.019	-.004	.023	,118**	-.064	.067	.051	.023	.024	-.015	,116**	,081*	--						
	Sig. (2-seitig)	.204	.925	.593	.911	.576	.001	.067	.060	.143	.507	.482	.675	.001	.044							
16) TM * Sleep (insomnia severity)	Pearson-Korrelation	.025	-,103**	-.034	-.005	-,095*	-,078*	-,184**	-.007	.019	.048	.002	-.010	-,065*	-,138**	-.036	--					
	Sig. (2-seitig)	.447	.001	.302	.867	.013	.025	.0	.845	.560	.138	.951	.761	.046	.0	.307						
17) TM * Sedentary behavior (hours/day)	Pearson-Korrelation	-.016	.037	-.009	.007	-.068	,074*	-.011	.0	-.024	-.020	-.004	.001	-.037	-,223**	,242**	.044	--				
	Sig. (2-seitig)	.619	.260	.786	.839	.078	.036	.749	.997	.475	.545	.895	.986	.264	.0	.0	.190					
18) TM * Alcohol Consumption	Pearson-Korrelation	.008	-.011	.017	-.003	.063	.060	.021	-.028	.002	.013	-.009	.015	-,101**	-.019	-.011	.042	.040	--			
	Sig. (2-seitig)	.803	.724	.591	.920	.095	.083	.523	.405	.954	.689	.788	.658	.002	.616	.744	.193	.222				
19) TM * Smoking	Pearson-Korrelation	.005	-.004	.021	.030	-.030	.028	.061	-.016	-.014	.042	-.049	.016	-.037	-.022	-,104**	,068*	.029	,362**	--		
	Sig. (2-seitig)	.871	.910	.517	.353	.428	.414	.061	.620	.665	.190	.133	.636	.258	.571	.003	.037	.389	.0			
20) TM * Pharmacological neuroenhancement	Pearson-Korrelation	-.008	-,144**	.005	.001	-.049	-.023	-.019	-.0	.018	.022	-.026	-.048	-.057	-.049	-,073*	,151**	.012	,251**	,248**	--	
	Sig. (2-seitig)	.819	.0	.882	.979	.210	.521	.567	.995	.579	.507	.429	.150	.087	.205	.040	.0	.719	.0	.0		
21) TM * Smoking marihuana	Pearson-Korrelation	.008	-,089**	-.024	.001	-.009	.034	.006	-.004	-.011	-.005	.017	-.025	-.059	-,083*	-.065	.051	-.005	,198**	,305**	,526**	--
	Sig. (2-seitig)	.815	.006	.451	.985	.821	.325	.846	.893	.737	.888	.595	.458	.070	.028	.060	.115	.887	.0	.0	.0	

**. Significant correlation on level p ≤ .01

*. Significant correlation on level p ≤ .05

For correlation, categorical variables were treated as metric.

Supplementary Table S4: Cross validation with an 80% random sample of the linear regression analysis of academic performance with stepwise inclusion of health behaviours and health behaviours' interaction with trait mindfulness.

	Model 1: covariates and health behaviours to predict academic performance												Model 2: covariates, health behaviours, trait mindfulness (TM), and interactions between health behaviours and TM to predict academic performance		
	Step 1: covariates (R^2 = 0.008*)			Step 2: big three health behaviours (R^2 = 0.021*)			Step 3: other health behaviours (R^2 = 0.015*)			Step 4: all health behaviours (R^2 = 0.027*)			(R^2 = 0.023)		
	B (SE)	*β*	*p*	B (SE)	*β*	*p*	B (SE)	*β*	*p*	B (SE)	*β*	*p*	B (SE)	*β*	*p*
Gender "female"	-.19 (.07)	-.09	.012	-.16 (.10)	-.07	.118	-.16 (.08)	-.08	.039	-.14 (.10)	-.06	.192	-.16 (.11)	-.07	.149
Gender "diverse"	.74 (.45)	.06	.104	-.15 (.62)	-.01	.823	.80 (.52)	.06	.125	.06 (.65)	<-.01	.921	-.03 (.66)	<-.01	.960
Gender "open"	-.26 (.29)	-.03	.373	-.14 (.38)	-.02	.724	-.45 (.35)	-.05	.199	-.82 (.46)	-.08	.080	-.78 (.47)	-.08	.101
Age	<-.01 (<.01)	-.03	.421	<-.01 (<.01)	-.02	.684	-.01 (.01)	-.04	.257	<-.01 (<.01)	-.02	.742	<-.01 (.01)	-.02	.730
Moderate-to-vigorous physical activity (minutes/week)				<.01 (<.01)	.06	.185				<.01 (<.01)	.06	.234	<.01 (<.01)	.07	.163
Fruit and vegetables consumption (portions/day)				-.03 (.02)	-.10	.033				-.04 (.02)	-.11	.014	-.04 (.02)	-.12	.018
Sleep (insomnia severity)				.14 (.05)	.13	.004				.13 (.05)	.12	.011	.12 (.05)	.11	.023
Sedentary behaviour (hours/day)							-.01 (.01)	-.03	.455	<-.01 (.02)	-.02	.680	<-.01 (.02)	<-.01	.908
Alcohol Consumption (AUDIT-C score)							-.02 (.05)	-.01	.756	-.04 (.07)	-.03	.553	-.02 (.07)	-.01	.804
Smoking (currently: no/yes)							.13 (.10)	.57	.195	.20 (.13)	.08	.127	.14 (.13)	.06	.277
Smoking marihuana (currently: no/yes)							-.18 (.14)	-.05	.207	-.03 (.16)	-.01	.836	<-.01 (.17)	<-.01	.974
Pharmacological neuroenhancement (past 12 months: no/yes)							.31 (.13)	.10	.019	.16 (.17)	.05	.342	.16 (.18)	.05	.361
Trait mindfulness													<-.01 (.09)	<-.01	.966
TM * Moderate-to-vigorous physical activity (minutes/week)													<.01 (<.01)	.03	.548

TM * Fruit and vegetables consumption (portions/day)	.04 (.04)	.05	.266
TM * Sleep (insomnia severity)	.06 (.12)	.03	.609
TM * Sedentary behaviour (hours/day)	<-.01 (.04)	<-.01	.983
TM * Alcohol Consumption	-.25 (.16)	-.08	.117
TM * Smoking	-.30 (1.09)	-.01	.782
TM * Pharmacological neuroenhancement	.21 (.52)	.02	.682
TM * Smoking marihuana	-.02 (.50)	<-.01	.967

Note:

*: $p < .05$

Model 1: step 1: $p = .025$; Step 2: $p = .015$; Step 3: $p = .019$; Step 4: $p = .017$

Model 2: $p = .070$

TM: trait mindfulness

References

1. Hagströmer M, Oja P, Sjöström M. The International Physical Activity Questionnaire (IPAQ): a study of concurrent and construct validity. Public Health Nutr 2006; 9(6):755–62.
2. Craig CL, Marshall AL, Sjöström M, Bauman AE, Booth ML, Ainsworth BE et al. International physical activity questionnaire: 12-country reliability and validity. Med Sci Sports Exerc 2003; 35(8):1381–95.
3. Aune D, Giovannucci E, Boffetta P, Fadnes LT, Keum N, Norat T et al. Fruit and vegetable intake and the risk of cardiovascular disease, total cancer and all-cause mortality-a systematic review and dose-response meta-analysis of prospective studies. Int J Epidemiol 2017; 46(3):1029–56.
4. Wang X, Ouyang Y, Liu J, Zhu M, Zhao G, Bao W et al. Fruit and vegetable consumption and mortality from all causes, cardiovascular disease, and cancer: systematic review and dose-response meta-analysis of prospective cohort studies. BMJ 2014; 349:g4490.
5. European Commission. European Health Interview Survey (EHIS wave 2): Methodological manual : 2013 edition. Luxembourg: Publications Office of the European Union; 2013. (Methodologies and working papers).
6. Gerber M, Lang C, Lemola S, Colledge F, Kalak N, Holsboer-Trachsler E et al. Validation of the German version of the insomnia severity index in adolescents, young adults and adult workers: results from three cross-sectional studies. BMC Psychiatry 2016; 16:174.
7. World Health Organization, Babor TF, Higgins-Biddle JC, Saunders JB, Monteiro MG. AUDIT: the alcohol use disorders identification test : guidelines for use in primary health care, 2nd ed.; 2001 [cited 2023 May 8]. Available from: URL: https://apps.who.int/iris/handle/10665/67205.
8. Barry AE, Chaney BH, Stellefson ML, Dodd V. Evaluating the psychometric properties of the AUDIT-C among college students. Journal of Substance Use 2015; 20(1):1–5.
9. Bush K, Kivlahan DR, McDonell MB, Fihn SD, Bradley KA. The AUDIT alcohol consumption questions (AUDIT-C): an effective brief screening test for problem drinking. Ambulatory Care Quality Improvement Project (ACQUIP). Alcohol Use Disorders Identification Test. Arch Intern Med 1998; 158(16):1789–95.

10. Töpritz K, Lohmann K, Gusy B, Farnir E, Gräfe C, Sprenger M. Wie gesund sind Studierende der Technischen Universität Kaiserslautern? Ergebnisse der Befragung 06/15. Berlin: Freie Universität Berlin.: Schriftenreihe des AB Public Health: Prävention und psychosoziale Gesundheitsforschung: Nr. 01/P16; 2016.

11. Ford JA, Pomykacz C. Non-Medical Use of Prescription Stimulants: A Comparison of College Students and their Same-Age Peers Who Do Not Attend College. Journal of Psychoactive Drugs 2016; 48(4):253–60.

12. Dietz P, Ulrich R, Dalaker R, Striegel H, Franke AG, Lieb K et al. Associations between physical and cognitive doping--a cross-sectional study in 2.997 triathletes. PLoS ONE 2013; 8(11):e78702.

13. Franke AG, Lieb K. Pharmakologisches Neuroenhancement und "Hirndoping" : Chancen und Risiken. Bundesgesundheitsblatt Gesundheitsforschung Gesundheitsschutz 2010; 53(8):853–9.

14. Sattler S. Cognitive Enhancement in Germany. In: Jotterand F, Dubljevic V, editors. Cognitive Enhancement. Oxford University Press; 2016. p. 159–80.

15. Dietz P, Soyka M, Franke AG. Pharmacological Neuroenhancement in the Field of Economics-Poll Results from an Online Survey. Front Psychol 2016; 7:520.

16. Franke AG, Bagusat C, Dietz P, Hoffmann I, Simon P, Ulrich R et al. Use of illicit and prescription drugs for cognitive or mood enhancement among surgeons. BMC Med 2013; 11:102.

17. Sjostrom M, Ainsworth BE, Bauman A, Bull FC, Hamilton-Craig CR, Sallis JF. Guidelines for data processing analysis of the International Physical Activity Questionnaire (IPAQ) - Short and long forms. In: ; 2005 [cited 2022 Oct 7]. Available from: URL: http://www.ipaq.ki.se.

18. Cheng HL. A simple, easy-to-use spreadsheet for automatic scoring of the International Physical Activity Questionnaire (IPAQ) Short Form; 2016.

19. Heller S, Tibubos AN, Hoff TA, Werner AM, Reichel JL, Mülder LM et al. Potential risk groups and psychological, psychosocial, and health behavioral predictors of pharmacological neuroenhancement among university students in Germany. Sci Rep 2022; 12(1):937.

20. Walach H, Buchheld N, Buttenmüller V, Kleinknecht N, Grossmann P, Schmidt S. Empirische Erfassung der Achtsamkeit - Die Konstruktion des Freiburger Fragebogens zur Achtsamkeit (FFA) und weitere Validierungsstudien. In: Heidenreich T, editor. Achtsamkeit und Akzeptanz in der Psychotherapie: Ein Handbuch. Tübingen: DGVT Deutsche Gesellschaft f. Verhaltenstherapie; 2004. p. 727–65.
